# Somatic mutations in the human brain: implications for psychiatric research

**DOI:** 10.1038/s41380-018-0129-y

**Published:** 2018-08-07

**Authors:** Masaki Nishioka, Miki Bundo, Kazuya Iwamoto, Tadafumi Kato

**Affiliations:** 1 Division for Counseling and Support, The University of Tokyo, Tokyo, Japan; 2 Department of Molecular Brain Science, Graduate School of Medical Sciences, Kumamoto University, Kumamoto, Japan; 3 PRESTO, Japan Science and Technology Agency, Saitama, Japan; 4 Laboratory for Molecular Dynamics of Mental Disorders, RIKEN Brain Science Institute, Saitama, Japan

**Keywords:** Genetics, Neuroscience, Psychiatric disorders, Genetics, Schizophrenia

## Abstract

Psychiatric disorders such as schizophrenia and bipolar disorder are caused by complex gene–environment interactions. While recent advances in genomic technologies have enabled the identification of several risk variants for psychiatric conditions, including single-nucleotide variants and copy-number variations, these factors can explain only a portion of the liability to these disorders. Although non-inherited factors had previously been attributed to environmental causes, recent genomic analyses have demonstrated that de novo mutations are among the main non-inherited risk factors for several psychiatric conditions. Somatic mutations in the brain may also explain how stochastic developmental events and environmental insults confer risk for a psychiatric disorder following fertilization. Here, we review evidence regarding somatic mutations in the brains of individuals with and without neuropsychiatric diseases. We further discuss the potential biological mechanisms underlying somatic mutations in the brain as well as the technical issues associated with the detection of somatic mutations in psychiatric research.

## Somatic mutation as a candidate mechanism for psychiatric disorders

Advancements in genetic technologies such as microarray analysis and massively parallel sequencing have enabled us to analyze genetic information at a genome-wide level. Several of these genetic studies have identified candidate risk genes for a variety of psychiatric disorders. For example, a large-scale genome-wide association study (GWAS) identified 108 genomic loci associated with schizophrenia using single-nucleotide polymorphism (SNP) microarray technology [1]. Additional studies have identified several copy-number variations (CNVs) associated with either schizophrenia [2–4] or autism spectrum disorder (ASD) [5]. Despite extensive genetic investigation, the SNPs and CNVs identified to date can only partially explain the liability to psychiatric disorders [6]. Even the 108 significant loci and 8 CNVs identified in the aforementioned large-scale studies can explain only 3.4% and 0.85% of the variance in liability to schizophrenia, respectively [1, 7]. The contribution of genomic features to ASD is greater than that to schizophrenia, with 6.02% of patients with ASD exhibiting known rare variants [8]. Even after considering the contribution of rare mutations, the total liability to these disorders cannot be explained.

**Fig. 1:**
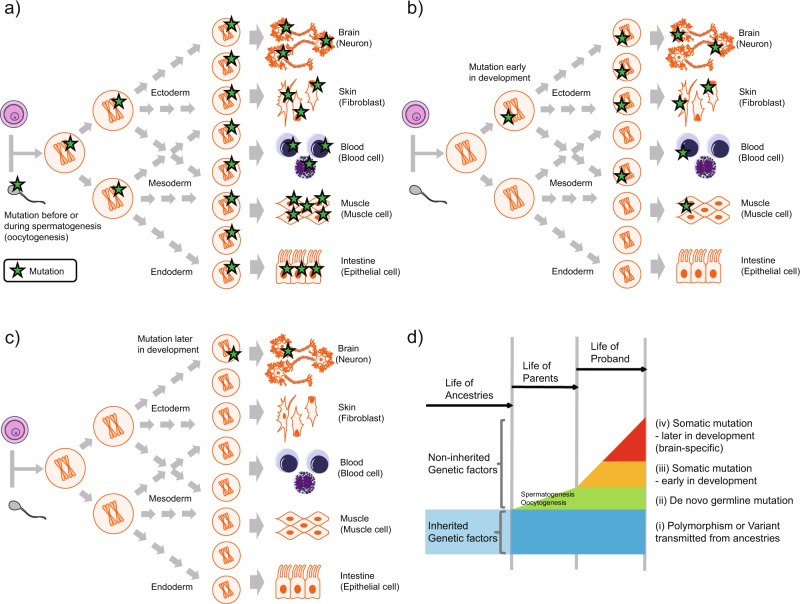
De novo or somatic mutations and developmental stage. **a** De novo mutations occur before or during spermatogenesis/oocytogenesis. Mutations in the sperm or oocytes descend to the fertilized egg and are shared among all tissues in the proband. The descendants of the proband will inherit this de novo germline mutation with a probability of 50%. **b** Somatic mutation occurring early in development, before the differentiation of somatic tissues. Mutations occurring early in development are shared among various tissues, but not all somatic cells or tissues, in the proband. The mutation exists in limited tissues or limited parts of each tissue. The descendants of the proband have a possibility of inheriting the somatic mutation, but with probability of <50%. **c** Somatic mutation occurring later in development, after the differentiation of somatic tissues. Mutations occurring after tissue differentiation are limited to a part of one tissue (brain, in this example) in the proband. The allele fraction of this type of somatic mutation is usually lower than that of somatic mutations occurring earlier. If the mutation is limited to the brain, the descendants of the proband will not inherit the somatic mutation. **d** A multi-layered scheme of genetic variants in a proband. (i) polymorphisms and variants transmitted from ancestries, (ii) de novo germline mutations, (iii) somatic mutations occurring early in development, and (iv) somatic mutations occurring later in development (brain-specific) from the viewpoint of a proband are illustrated with a time-axis. The polymorphisms and variants transmitted from ancestries are inherited genetic factors, but the other three mutation types are non-inherited genetic factors. These four types of germline or somatic variants (mutations) would have an additive effect on the individual phenotype. Somatic mutations (iii and iv) are the main focus of this review

Although the remaining liability to psychiatric disorders has classically been attributed to environmental factors, recent psychiatric research has focused on the role of de novo mutations, which represent a type of non-inherited genetic factor. De novo mutations occur prior to fertilization, before or during spermatogenesis/oocytogenesis (Fig. 1a). Some de novo mutations occurring before spermatogenesis/oocytogenesis are derived from genomic chimerism in either parent, which can be detected in a part of the somatic tissues of the parent [9–12]. In contrast, de novo mutations occurring during spermatogenesis/oocytogenesis cannot be detected in the tissues of the parents, except for in a limited number of germ cells. Trio analyses have revealed that de novo mutations in *SETD1A*, *CHD8*, and other critical variants are associated with an increased risk of multiple psychiatric disorders [13–17]. Large case–control studies have validated these findings regarding *SETD1A* and *CHD8* in patients with schizophrenia and ASD, respectively [18, 19].

In addition to germline de novo mutations, somatic or postzygotic mutations may occur following fertilization. Following such mutations, the genome in each somatic cell is not completely identical in one individual. Somatic mutations have also been well characterized as a pathological mechanism associated with cancer [20], and as an adaptive physiological mechanism associated with somatic rearrangement of immunoglobulin genes [21]. Cancers are caused by somatic mutations in key-driver genes in a specific tissue, and numerous additional somatic mutations may accrue with advancement. In addition to cancerous tissues, recent genomic studies have systematically identified somatic mutations at the genome-scale in non-cancerous human tissues [22–26]. Furthermore, some mutations originally labeled as germline de novo mutations have subsequently been identified as somatic mutations that occurred after fertilization in the children [27], or prior to spermatogenesis/oocytogenesis in the parents [9].

Several human diseases are known to result from somatic mutations [28], and accumulating evidence indicates that somatic mutations may explain in part the liability to psychiatric disorders [29–32]. Such mutations can be observed in various tissues during the early developmental period, including peripheral tissues (e.g., blood cells) as well as brain cells (Fig. 1b). In contrast, somatic mutations that occur following differentiation exist within a limited region of a single tissue type (e.g., brain), and thus can be detected only in that tissue (Fig. 1c). Somatic mutations occur due to environmental insults, including inflammation and oxidative stress (described below), as well as stochastic changes during development.

While polymorphisms and the variants transmitted from ancestries are inherited genetic factors, the other three mutation types of de novo and somatic mutations illustrated in Fig. 1a–c are non-inherited genetic factors. Nonetheless, these all four types of germline and somatic variants (mutations) likely have an additive effect on the individual phenotype (Fig. 1d). For example, research has indicated that germline de novo mutations and inherited variants additively contribute to the risk for ASD [33, 34]. In principle, mutations resulting in embryonic lethality or severe congenital diseases cannot exist in the germline genome, although they may exist as somatic mutations, possibly resulting in relatively less severe physiological consequences. Previous studies regarding epileptic encephalopathy have revealed that single somatic mutations of *PCDH19* result in less severe pathology than de novo mutations of the same gene [35, 36].

**Fig. 2:**
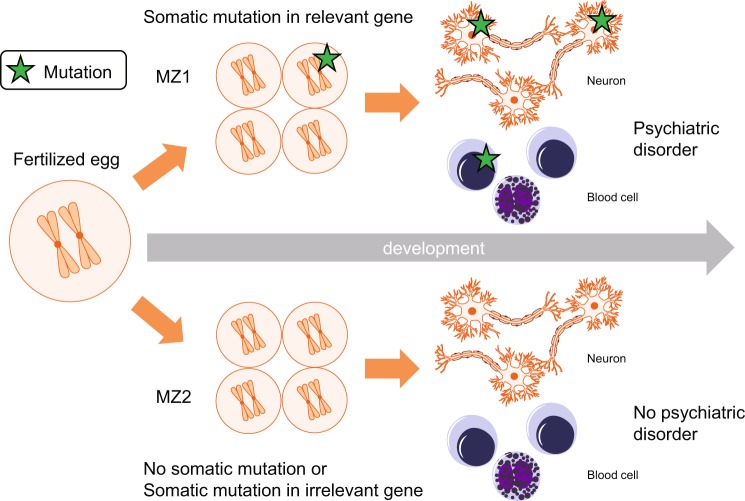
Somatic mutation model explaining phenotypic differences between monozygotic (MZ) twins. MZ twins have identical genomes at the time of fertilization, but somatic mutation profiles diverge after fertilization. Somatic mutations during development may underlie phenotypic differences between the twins, including discordant risk for psychiatric disorders. In this illustrated model, MZ1 has somatic mutations in the relevant genes in development, which are shared between the neurons and blood cells, and has a psychiatric diagnosis. MZ2 has no somatic mutations in the relevant genes does not have a psychiatric diagnosis

Polymorphisms and the variants transmitted from ancestries and de novo mutations, shared between monozygotic twins, contribute to the heritability estimated from studies of twins [37]. However, somatic mutations not shared between monozygotic twins do not directly contribute to the heritability, but affect the liability to psychiatric disorders in patients, by altering biological pathways similar to those affected by the germline mutations (Fig. 2). somatic mutations may contribute to a part of the total variation in liability that has been classically considered as being related to environmental factors.

The estimated rate of de novo mutations is 1–1.5 × 10^−8^ per nucleotide per generation [10, 38, 39]. Somatic mutations may be more common than de novo mutations. Assuming a conservative estimate of 2.8 substitution mutations per cell per cell division [24] and symmetrical divisions in development, 86 billion neurons [40] would have gone through at least 36 divisions, thus resulting in a minimum of 100 single-nucleotide variants (SNVs) in one neuron. In fact, neurons likely undergo many more cell divisions, and mutation within neural tissues occurs via mechanisms other than replication errors during cell division (described below). In addition, other types of mutations (e.g., structural variants) may occur, increasing the number of mutational events beyond this minimum estimation.

Previous research groups have published excellent reviews that organize recent knowledge regarding somatic mutation in the human brain from a neurological/neuroscientific perspective [30–32]. In the present report, we review recent evidence regarding somatic mutations in the human brain focusing on the implications of these findings for psychiatry as well as technical issues associated with genomic analysis. We begin by comparing somatic mutations in the brains of individuals without neuropsychiatric diseases (Table 1) to those in individuals with neuropsychiatric diseases (Table 2). We then discuss the potential biological mechanisms underlying somatic mutations in the brain, as well as technical issues for future psychiatric research. Although somatic mutations in mitochondria also have a critical role in psychiatric disorders, those studies were reviewed elsewhere [41, 42], and thus we have focused on somatic mutations in genomic DNA.

**Table 1:** Genomic analysis of somatic mutations in the brains of individuals without neuropsychiatric diseases

Mutation type	Brain region	Cell type	References
SNV	Frontal cortex	Single neuron	Lodato et al. [44]
	Fetal frontal cortex	Clonally cultured single cell	Bae et al. [43]
	Fetal parietal cortex	Clonally cultured single cell	Bae et al. [43]
	Fetal basal ganglia	Clonally cultured single cell	Bae et al. [43]
	Prefrontal cortex	Single neuron	Lodato et al. [45]
	Hippocampus	Single neuron	Lodato et al. [45]
	Frontal cortex	Bulk	Nishioka et al. [52]
	Cerebellum	Bulk	Nishioka et al. [52]
CNV	Frontal cortex	Single neuron Single iPS-derived neuron Single iPS-derived neural precursor cell	McConnell et al. [53]
	Frontal cortex	Single neuron	Cai et al. [54]
MEI (LINE-1, Alu)	Hippocampus	Bulk	Baillie et al. [65]
	Caudate nucleus	Bulk	Baillie et al. [65]
MEI (LINE-1)	Frontal cortex	Single neuron	Evrony et al. [68]
	Caudate nucleus	Single neuron	Evrony et al. [68]
	Frontal cortex	Single neuron	Evrony et al. [69]
	Hippocampus	Single neuron Single glia	Upton et al. [66]
	Caudate nucleus	Single neuron Single glia	Upton et al. [66]
Aneupoloidy	Frontal lobe	Single neuron	Knouse et al. [56]
	Frontal cortex	Single neuron	van den Bos et al. [121]
	Cortex	Neuron (FISH) Non-neuron (FISH)	Rehen et al. [55]
	Hippocampus	Neuron (FISH) Non-neuron (FISH)	Rehen et al. [55]

*SNV* single-nucleotide variant, *CNV* copy-number variation, *MEI* mobile element insertion, *LINE-1* long interspersed nuclear element-1, *FISH* fluorescence in situ hybridization

**Table 2:** Somatic mutations in patients with neuropsychiatric diseases

Disease/disorder	Implicated gene	Mutation type	Sample	References
Postmortem brain				
Hemimegalencephaly	PIK3CA, AKT3, MTOR	SNV	The affected brain region	Poduri et al. [95] Lee et al. [92] D’Gama et al. [93] Jansen et al. [94]
	1q trisomy/tetrasomy	CNV	The affected brain region	Poduri et al. [95] Cai et al. [54]
Cortical dysplasia type II	MTOR	SNV	The affected brain region	Lim et al. [97] Nakashima et al. [98] Mirzaa et al. [96]
Sturge–Weber syndrome	GNAQ	SNV	Brain	Shirley et al. [99] Nakashima et al. [100]
Huntington’s disease	HD	CAG repeat	Striatum	Kennedy et al. [103]
	HD	CAG repeat	Frontal cortex Cerebellum	Swami et al. [104]
Rett syndrome	LINE1 copy number	MEI	iPS-derived NPC	Muotri et al. [114]
Ataxia telangiectasia	LINE1 copy number	MEI	Hippocampal neuronal nuclei	Coufal et al. [113]
Cockayne syndrome	Global SNV increase	SNV	Prefrontal cortex neuron	Lodato et al. [45]
Xeroderma pigmentosum	Global SNV increase	SNV	Prefrontal cortex neuron	Lodato et al. [45]
Alzheimer’s disease	PSEN1	SNV	Cerebral cortex	Beck et al. [118]
	MAPT, PSEN2	SNV	Entorhinal cortex	Sala Frigerio et al. [116]
	Many (not validated)	SNV	Hippocampus	Parcerisas et al. [119]
	APP copy-number increase	CNV	Prefrontal cortex Cerebellum	Bushman et al. [117]
	No different aneuploidy	Aneuploidy	Frontal cortex	van den Bos et al. [121]
	Chromosome 21 loss/gain	Aneuploidy	Cerebral cortex	
	Excess aneuploidy	Aneuploidy	Entorhinal cortex	
			Occipital cortex	
Autism spectrum disorder	CACNA1C, SCN1A, SETD2	SNV	Prefrontal cortex Cerebellum	D’Gama et al. [115]
Schizophrenia	LINE-1 copy number	MEI	Cortex neuronal nuclei Cortex non-neuronal nuclei iPS-derived neurons	Bundo et al. [89]
	LINE-1 copy number	MEI	Dorsolateral prefrontal cortex	Doyle et al. [112]
	Deletions in PRKRA and others	CNV	Prefrontal cortex Cerebellum	Kim et al. [108]
	1p36.21, 1p13.3	CNV	Striatum	Sakai et al. [109]
	Chromosome 1 loss/gain	Aneuploidy	Cortex (Brodmann area 10)	Yurov et al. [110]
Peripheral tissues				
Hemimegalencephaly	PIK3CA	SNV	Blood Saliva Buccal swab	Riviere et al. [105]
Megalencephaly	PIK3CA	SNV	Blood	Mirzaa et al. [96]
Double cortex syndrome	DCX, LIS1	SNV	Blood	Jamuar et al. [106]
Periventricular nodular heterotopia	FLNA	SNV	Blood	Jamuar et al. [106]
Pachygyria	TUBB2B	SNV	Blood	Jamuar et al. [106]
Rett syndrome	MECP2	Small deletion	Peripheral blood lymphocytes	Clayton-Smith et al. [125]
Autism spectrum disorder	KMT2C, NCKAP1, MYH10, and others	SNV	Blood	Freed et al. [122]
	MFRP,MYO9B, PTK7, TANC2, MEGF11, and others	SNV	Blood	Dou et al. [11]
	KLF16, MSANTD2, SCN2A, HNRNPU, SMARCA4, and others	SNV	Blood	Lim et al. [123]
	CHD2, CTNNB1, KMT2C, SYNGAP1, RELN, and others	SNV	Blood	Krupp et al. [12]
Monozytotic twin				
Darier disease	ATP2A2	Small deletion	Blood (MZ)	Sakuntabhai et al. [130]
Van der Woude syndrome	IRF6	SNV	Blood (MZ)	Kondo et al. [131]
Dravet syndrome	SCN1A	SNV	Lymphocytes (MZ) Hair-follicle cells (MZ) Cheek cells (MZ) Fibroblasts (MZ) Olfactory neuroepithelium (MZ)	Vadlamudi et al. [132]
Neurofibromatosis type 1	NF1	SNV	Blood (MZ) Buccal swab (MZ) Urine (MZ)	Vogt et al. [133]
Parkinson-related diseases	31 loci	CNV	Blood (MZ)	Bruder et al. [136]
Fragile X syndrome (severity)	FMR1	CGG repeat	Blood (MZ)	Helderman-van den Enden et al. [135]
Gender dysphoria	FBXO38, SMOC2, TDRP	SNV	Blood lymphocytes (MZ)	Morimoto et al. [134]
Delusional disorder	ABCC9	SNV	Blood (MZ)	Nishioka et al. [137]

Note that relevancy of the implicated genes to each disease/disorder differs among the listed studies

*SNV* single-nucleotide variant, *CNV* copy-number variation, *MEI* mobile element insertion, *LINE-1* long interspersed nuclear element-1, *FISH* fluorescence in situ hybridization

## Somatic mutations in the brains of individuals without neuropsychiatric diseases

### SNVs

One neuronal progenitor cell in the fetal cortex has ~200–400 somatic SNVs [43], while one mature neuron in the adult cortex has ~1000–2500 somatic SNVs [44, 45]. These estimations are based on whole-genome sequencing (WGS) of single-cell genomes after clonal cell proliferation or whole-genome amplification (WGA). SNVs in a single neuron in the prefrontal cortex and dentate gyrus increase by ~23 and ~40 per year, respectively [45]. The accumulation rate in dentate gyrus neurons is twofold higher than that in neurons of the prefrontal cortex, probably owing to the difference in the rate of neurogenesis. Some mutations in single neurons are also detected in other brain regions with various allele fractions [43, 44]. The allele fractions in certain brain regions are postulated to be correlated with the time point of mutation occurrence, such that larger allele fractions may reflect mutation during the earlier stages of development. Bae et al. [43] provided an estimation of 5.1 SNVs/day/single progenitor cell during neurogenesis, 8.6 SNVs/division/progenitor cell, and 1.3 SNVs/division/daughter cell in the early human embryo. Neurogenesis is associated with a higher mutation rate than early embryogenesis, although frequency of adult neurogenesis in the human brain is under active discussion [46].

The cytosine (C) to thymine (T) transition is a prominent mutation type in neuronal somatic SNVs [43–45]. The frequency of approximately 80% of C>T [44] is higher than the frequency observed in germline mutations [10, 47], although a part of the detected C>T may be derived from experimental artifacts [48]. The C>T transition is enriched at 5-methylated cytosines (5mC) in CpG and CpH dinucleotides, suggesting that these mutations are derived from DNA methylation/demethylation [44]. With aging, the C>T fraction decreases, while the cytosine to adenine (C>A) and T>C fractions increase in the cortex [43, 45]. The dentate gyrus neuron is one exception, where the C>T fraction increases with aging, probably due to neurogenesis [45]. The C>A transversion is probably induced by oxidative DNA damage (see below) [43, 45]. Lodato et al. [45] inferred that the T>C increase is linked to fatty-acid oxidation DNA damage.

The observed somatic mutations were enriched in neurodevelopment-related genes and strands undergoing active transcription. The higher length of the neuron-expressed genes [49–51] may be a reason for mutational susceptibility. Somatic mutations in mature neurons likely occur during active transcription [44], while somatic mutations in fetal neurons are less abundant in the genomic regions with histone marks of fetal brains and embryonic stem cells [43]. The genomic regions susceptible to mutation may differ depending on the developmental stage. The bias of somatic mutations toward neurodevelopmental genes in mature neurons is indicative of their relevance to psychiatric disorders.

In a recent study, we explored somatic mutations in human brain tissue from three healthy individuals via WGS at a depth of ~100×. Allele fractions were assayed and validated via target amplicon sequencing (TAS), ranging from 0.5 to 14% in bulk tissue obtained from the cortex, cerebellum, and liver [52]. The somatic mutations exhibited considerable spatial diversity; some mutations were observed in the brain but not the liver, while others were observed in the cortex but not the cerebellum. In accordance with the findings of previous researchers, we also observed a greater frequency of somatic mutations and C>T bias in neuron-expressed genes.

### CNVs and aneuploidy

Somatic CNVs have also been reported at the single-cell level. McConnell et al. [53] assayed somatic CNVs in the genomes of individual neurons using low-depth WGS and microarray analysis following WGA with degenerate oligonucleotide-primed PCR (DOP-PCR). Forty-five of 110 (40.9%) single neurons in the frontal cortex tissue from three healthy individuals exhibited somatic CNVs with lengths ranging from 2.9 to 75 Mb. In these somatic CNVs, deletions were more frequent than duplication. In a similar study, Cai et al. [54] assayed somatic CNVs in the genomes of individual neurons via low-depth WGS using two methods of WGA, multiple displacement amplification (MDA) with phi29 DNA polymerase and DOP-PCR. Five percent of 82 neurons from three healthy men exhibited aneuploidy (aberrant haploid sets). The authors estimated that the average single neuron had 3.4 CNVs with an approximate size of 18 Mb each. Neurons exhibited more deletions and single-cell-specific CNVs than lymphoblastoid cells, likely reflecting a low rate of proliferation in neurons. Notably, 98% of the neuronal CNVs were deletions, while only around 50% of CNVs in the lymphoblastoid cells were deletions in this study. Among the somatic CNVs identified was a 2.9 Mb duplication at 15q13.2-13.3, which has been associated with schizophrenia [3] and ASD [5]. One interpretation is that 15q13.2-13.3 is vulnerable to copy-number alterations in neurons as well as germlines.

Several research groups have identified somatic aneuploidy in the human brain. Rehen et al. [55] reported that 4% of the neurons and glial cells exhibited somatic aneuploidy in contrast to 0.6% of the white blood cells. This estimation was based on microscopic observation following fluorescence in situ hybridization (FISH) analyses, which revealed that chromosomal deletion and duplication occurred at a ratio of 1:1, likely due to unbalanced separation of chromosomes during mitosis. Single-cell WGS (depth: 0.1×) experiments by Knouse et al. [56] demonstrated that ~2.2% of 89 neurons from the frontal lobes of four individuals without neuropsychiatric diseases exhibited aneuploidy. Based on the results of FISH and single-cell WGS analyses, the aneuploidy rate of neurons should be 2–4%. Previous studies have identified tetra-ploidy in mouse pyramidal neurons of cortical layer 5 [57], encouraging future investigations of an equivalent phenomenon in human neurons.

### Mobile elements

Mobile elements occupy ~45% of the human genome [58]. Long interspersed nuclear element-1 (LINE-1) is one of the major mobile elements, exhibiting active transposition abilities and occupying 17% of the human genome. The LINE-1 sequence can proliferate in the human genome through the following processes called retrotransposition: (i) transcription of mRNA from an internal promoter; (ii) translation of LINE-1 mRNA, producing a LINE-1 RNA-protein complex with endonuclease and reverse transcriptase activity; (iii) reverse-transcription to DNA and concurrent insertion into the human genome [59]. Several heritable genetic diseases such as hemophilia [60] are known to result from germline LINE-1 retrotransposition [61]. The retrotransposition activity of LINE-1 is usually suppressed by epigenetic systems, including DNA methylation and histone modification. However, LINE-1 retrotransposition activity has been identified in the neuronal genome of both humans and mice [62–64].

The rate of somatic retrotransposition in the human brain is under active investigation. Baillie et al. [65] observed somatic retrotransposition of LINE-1 and Alu in bulk tissue obtained from the human hippocampus and caudate nucleus. Somatic retrotransposition was detected using retrotransposon capture sequencing (RC-Seq), in which retrotransposons in the genome were massively sequenced following capture. The insertion sites of retrotransposons were identified by flanking genomic sequences. The authors detected 13,700 and 7700 putative somatic retrotranspositions of LINE-1 and Alu, respectively, in three individuals. Insertion enrichment was also observed in exons and synapse-related genes. Applying RC-Seq to neurons and glia, Upton et al. [66] reported LINE-1 insertion sites in single cells, using WGA by multiple annealing and looping-based amplification cycles (MALBAC) [67]. After Sanger sequencing validation, they estimated that the average somatic LINE-1 insertions per single cell are ~13.7, 6.5, and 10.7 in hippocampal neurons, hippocampal glia, and cortical neurons, respectively. These estimations were calculated based on experiments involving 92 hippocampal neurons from four individuals, 22 hippocampal glia from three individuals, and 35 cortical neurons from three individuals.

Evrony et al. detected LINE-1 insertions in single neurons using two independent approaches after WGA of single-neuron genomes with MDA [68, 69]. The first approach, L1Hs-seq [70], was used to comprehensively amplify and massively sequence genomic regions around the 3′ end of L1Hs. LINE-1 insertion sites were determined based on the sequences adjacent to the L1Hs 3′ end [68]. Among the various LINE-1 subfamilies, L1Hs is the only group that exhibits retrotransposition activity in humans. The authors examined a total of 300 neurons from the frontal lobe and caudate nucleus of three healthy individuals. Sanger sequence validation suggested that ~0.07 new insertions per neuron occurred in this population. In the second approach, the authors performed bioinformatics analysis of WGS data obtained at a depth of 40× [69]. The sequence reads containing the LINE-1 sequence were extracted from the raw WGS data, and the insertion position of LINE1 was determined from the adjacent sequence. The second approach detected two somatic LINE-1 insertions in 16 single neurons obtained from one healthy man. The insertion sites and allele fractions of new insertions were validated and quantified using ddPCR, with allele fractions ranging from 0.04 to 1.7% in various brain regions.

As previously mentioned, estimates of new LINE-1 insertion in single neurons range from 0.07 to 13.7. Evrony et al. [71] reanalyzed the data of Upton et al., highlighting that most of the new somatic insertions reported were artifacts due to WGA. Stringent reanalysis of the data led them to estimate that the actual rate of new insertions was around 0.2 per single neuron. Thus, the rate of LINE-1 insertions in human neurons remains a subject of active discussion.

## Biological mechanisms underlying somatic mutations in the brain

Somatic mutations associated with cancer are thought to derive primarily from cell proliferation [20]. As neural tissue exhibits little to no proliferative capacity, the mechanisms underlying somatic mutation in the brain should be different from those associated with cancer.

Previous research has indicated that C>T transitions account for ~80% of somatic SNVs in single neurons [44]. Although some of these C>T transitions were likely derived from artificial C>U deamination due to cell lysis during the preparation of single-cell samples [48], we also observed the same bias for C>T transition following a WGS analysis of bulk brain tissues, which were free from deamination during cell lysis [52]. Neurons have a characteristic 5mC at non-CpG sites and a relatively greater amount of 5-hydroxymethylcytosine (5hmC) than other tissues [72, 73]. Moreover, 5hmC is an intermediate product during active demethylation of 5mC to C, and previous studies have suggested that this process, including base excision repair, is susceptible to mutation [74–76]. However, one study reported that 5hmC was associated with an ~53% decrease in the frequency of C>T mutations at CpG sites when compared with 5mC [77]. It is also possible that AID/APOBEC cause in vivo cytosine deamination of mC to T [78], although this process is reported to be chemically disfavored [79].

Neural activity induces double-stranded breaks in genomic DNA [80], and mutations can occur during the repair process. Several studies have suggested that these double-stranded breaks are enriched in genes associated with neural or synaptic activity [81, 82]. Double-stranded breaks represent one candidate mechanism underlying the development of somatic mutations in the human brain.

Although oxidative damage is not restricted to the brain, the brain accounts for ~20% of all oxygen consumption in the human body, thus sustaining a greater amount of oxidative damage than other organs. Oxidative damage is estimated to cause ~1000 single-stranded breaks per cell per day [83]. Oxidative damage produces 8-oxoguanine, which occasionally incorporates adenine as an incorrect complementary base. If DNA repair systems fail to recover the original pair (guanine and cytosine), the incorrectly incorporated adenine incorporate thymine as a correct complementary base, resulting in C>A transversions [84]. Research has revealed that oxidative damage is enriched in the promoter regions of genes associated with synaptic plasticity, vesicular transport, calcium signaling, and mitochondrial function, and that such damage reduces the expression of these genes [85]. Impaired mitochondrial function may also contribute to DNA damage by increasing reactive oxygen species or reducing the ATP available for DNA repair [86]. Oxidative damage to DNA in the human brain accumulates with age, particularly in the mitochondria [87], and has been shown to be associated with neurodegeneration [88].

The mechanism underlying retrotransposition in the human brain remains largely unknown. While epigenetic modification is assumed to suppress retrotransposon transcriptional activity [59], the mechanisms underlying retrotransposition in the human brain require further investigation [62–64]. Our data suggested that inflammatory stress during the fetal period causes somatic retrotransposition in a polyinosinic-polycytidylic acid (polyI:C) mouse model [89], suggesting that environmental factors lead to somatic retrotransposition, likely through neuroinflammation.

Retrotransposons or repeat sequences can induce non-homologous recombination of DNA [59], resulting in genome instability and somatic CNVs during development. In addition, LINE-1 can induce deletion between LINE-1 regions via endonuclease activity even without transcription or new insertion [90]. One study suggested that circular DNA fragments with a length of 200–400 bp are enriched in the adult mouse brain, and that somatic microdeletions in brain genomes arise from the excision of small circular DNAs [91]. However, the mechanisms underlying the generation of somatic SNVs, CNVs, and retrotransposition remain to be fully elucidated.

## Somatic mutations in brain malformations and neurodegenerative disorders

Several studies have identified somatic mutations in the known risk genes for certain brain malformations. In many cases, potential somatic mutations have been identified in brain regions exhibiting local anatomical changes.

Hemimegalencephaly refers to a disease in which one cerebral hemisphere grows to be larger than the other. Several studies utilizing whole-exome sequencing (WES) or TAS have observed somatic SNVs in *PIK3CA*, *AKT3*, or *MTOR* in the overgrown brain region (dissected during surgical treatment) [92–96]. Lee et al. [92] reported somatic mutations with allele fractions of 8–40% in the affected regions of 6 out of 20 patients with hemimegalencephaly following mass spectrometry analysis. Other groups have reported an allele fraction within a similar range or higher [93–95]. Single-cell CNV analysis revealed 1q tetrasomy in 20% of 76 single cells (46 neurons and 30 non-neurons) obtained from the affected brain region of one patient with hemimegalencephaly [54]. The same group observed somatic 1q trisomy in two other patients with hemimegalencephaly [95]. Considering that *AKT3* is on 1q, these findings indicate that somatic CNVs of 1q may also cause hemimegalencephaly.

Cortical dysplasia with epilepsy is another example of a brain malformation caused by somatic mutations [96–98]. Lim et al. [97] identified somatic mutations in *MTOR* in the affected brain region of patients with cortical dysplasia type II and epilepsy. They detected putative somatic mutations using WES at a depth of 400–700×, validating and quantifying the candidate mutation via TAS. Twelve of 77 patients had somatic mutations in *MTOR* with allele fractions of 1.3 to 12.6% in the affected regions. Additional experiments in model mice demonstrated the causality from *MTOR* somatic mutation to epilepsy, as well as the efficacy of rapamycin for the treatment of epilepsy in such cases. Nakashima et al. [98] detected somatic SNVs in *MTOR* in the affected brain regions of six out of 13 patients with cortical dysplasia type IIb. Somatic SNVs were detected via WES and TAS, and allele fractions of somatic SNVs in *MTOR* were determined as 1.54 to 9.31% or 1.45 to 5.51% via TAS or ddPCR, respectively. The associations between somatic mutation in the relevant genes and hemimegalencephaly/cortical dysplasia have been independently reported by several groups and are thus considered reliable.

Somatic mutations in *GNAQ* (c.548G>A, p.Arg183Glu) have been reported in patients with Sturge–Weber syndrome (a rare congenital neurological disorder characterized by seizures, mental retardation, cerebral malformations, and other symptoms). Shirley et al. [99] reported an allele fraction of ~11.15% in the brains of 15 of the 18 recruited patients, while Nakashima et al. [100] reported an allele fraction of approximately 8.94% in the brains of 12 of the 15 recruited patients.

Triplet repeat expansion is thought to be a causative mutation for several neurodegenerative diseases such as Huntington’s disease and fragile X syndrome [101]. Triplet repeats are unstable in somatic cells, including those of the brain [102]. Somatic triplet repeat expansion has been identified in the brains of patients with Huntington’s disease [103], particularly in brain regions with aggressive neurodegeneration, and has been correlated with disease onset at an early age [104]. Patients with Cockayan syndrome and Xeroderma pigmentosum, genetic disorders with neurological symptoms caused by defects in the DNA damage repair system, have a 2.5-fold higher rate of neuronal somatic mutations than healthy individuals [46]. C>A transitions are characteristic of these patients, indicating oxidative DNA damage.

Intriguingly, somatic mutations in the relevant genes have been identified not only in the affected brain regions, but also in peripheral tissues. Somatic mutations in *PIK3CA* were observed in the blood or saliva samples from 10 patients with hemimegalencephaly, with allele fractions ranging from 1 to 43% when examined via TAS [105]. Somatic SNVs in blood cells have also been observed in patients with other severe brain malformations such as double cortex syndrome, periventricular nodular heterotopia, and pachygyria. Jamuar et al. [106] selected the known risk genes for these brain malformations and performed TAS on the blood samples obtained from 158 patients. Eight of these 158 patients exhibited somatic SNVs in *DCX, LIS1, TUBB2B*, or *FLNA*. The allele fraction ranged from 5 to 35%, suggesting that these mutations occurred early in development and likely existed in the neural tissues. Such somatic mutations likely explain the pathogenesis of certain brain malformations.

## Somatic mutations and psychiatric disorders: postmortem brain analyses

Psychiatric disorders are often associated with fewer anatomical changes than brain malformations, making it relatively difficult to explore somatic mutations associated with these conditions. In one early study, one individual who had died by committing suicide exhibited a CNV specific to pons, although no clinical psychiatric data were presented [26]. Telomere length is generally variable in somatic cells. A characteristic reduction in telomere length has been observed in the hippocampus of patients with major depressive disorder [107].

In schizophrenia, WGS experiments by Kim et al. [108] revealed somatic deletions in *BOD1*, *CBX3*, *PRKRA*, *MIR548N*, *MRPL42*, *SUCLG2*, *TDG*, and another intergenic region in the prefrontal cortex, cerebellum, and white matter of three patients with schizophrenia. These somatic deletions were validated via Sanger sequencing, the lengths of which ranged from 466 to 5604 bp. Of note, similar somatic deletions of approximately 500 bp were detected in chromosome 2 (*PRKRA* and *MIR548N*) in samples from two independent patients, indicating that this region exhibits vulnerability to mutation. Two color microarray analysis of striatal tissue samples from 48 patients revealed gene dosage loss at 1p36.21 and 1p13.3, and these results were further validated using qPCR [109]. Another study reported excess aneuploidy in postmortem brain samples from patients with schizophrenia [110]. Taken together, these findings indicate the relevance of somatic CNVs in schizophrenia.

Previously, we reported a LINE-1 copy number increase in the postmortem brains (frontal cortex) of patients with schizophrenia in two independent cohorts [89]. The copy-number increase of LINE1 was characteristic of the genomes from isolated neuronal nuclei [111], suggesting somatic retrotransposition in neurons in schizophrenia. Increased LINE-1 copy number has also been observed in animal models of schizophrenia and neurons differentiated from induced pluripotent stem cells derived from patients. Of note, LINE-1 insertions were enriched in neuron-expressed genes. Doyle et al. [112] independently confirmed these results, demonstrating increased LINE-1 insertion in genes associated with synaptic function and schizophrenia in the postmortem dorsolateral prefrontal cortex of 36 patients with schizophrenia. LINE-1 copy number increases have also been observed in the postmortem brains of patients with Rett syndrome and ataxia telangiectasia [113, 114]. However, further studies are required to determine the precise site of new insertion during somatic LINE-1 retrotransposition, and to elucidate the association between LINE-1 retrotransposition and schizophrenia.

Several somatic SNVs have been associated with ASD. Five somatic SNVs, including three somatic SNVs on *CACNA1C*, *SCN1A*, and *SETD2*, were detected in the postmortem brains of five patients with either ASD or fragile X syndrome [115]. As these genes are well-established candidate risk genes for ASD, these findings indicate that somatic mutations may be associated with the development of ASD.

The risk genes and brain regions associated with Alzheimer’s disease (AD) have been relatively more elucidated than those associated with other psychiatric disorders, and several research groups have identified somatic mutations in the brains of patients with AD [116–119]. Sala Frigerio et al. [116] explored somatic mutations in the entorhinal cortex of 72 patients with AD and 58 healthy controls via TAS of the *APP*, *PSEN1*, *PSEN2*, and *MAPT* genes. The entorhinal cortex exhibits AD pathology in the very early stages of the disease, and *APP*, *PSEN1*, and *PSEN2* are the strongest known risk genes for AD. *MAPT* in particular is known to involve tangle formation. The authors identified three somatic mutations in *MAPT* in patients with AD and *PSEN2* in healthy individuals using ultra deep TAS validation, with allele fractions ranging from 0.7 to 1.6% in the entorhinal cortices. Bushman et al. [117] identified up to 12 copies of *APP* in the cortical genome of patients with AD via single-cell qPCR. They also reported that cortical nuclei from patients with AD exhibited an average DNA content increase of ~8%, relative to controls. Various groups have also observed excess aneuploidy in postmortem brain samples from patients with AD using FISH [120]. However, one single-cell WGS study reported no characteristic aneuploidy associated with AD [121]. In this previous study, the authors analyzed 30–130 single neurons obtained from the frontal cortex of each of the 10 patients with AD and six healthy controls. Three patients with AD exhibited aneuploidy in 1.7–2.7% of the cells, while one of the control individuals exhibited aneuploidy in 5.6% of the cells. The authors concluded the absence of strong evidence for common aneuploidy in normal and Alzheimer’s disease neurons.

## Somatic mutations and psychiatric disorders: analyses of peripheral tissue

Four groups have investigated somatic mutations using WES data for blood samples obtained from large cohorts of families with ASD (Simons Simplex Collection) [11, 12, 122, 123]. These WES data were originally obtained to investigate germline mutations. Lim et al. [123] added a large number of samples, analyzing WES data from ~6000 families. These four groups detected several hundred somatic mutations in blood cells, including mutations of *CHD2*, *RELN*, *SCN2A*, *SYNGAP1*, and other known ASD risk genes. Seven to 22 percent of the mutations originally identified as germline de novo mutations [14, 33] were eventually discovered to be postzygotic somatic mutations. Accumulating evidence indicates that the somatic mutations detected in these blood samples contribute an estimated 3–5% to ASD diagnosis [11, 12, 122].

Further analyses of blood samples from families with ASD have revealed that somatic SNVs are enriched on the antisense strand, indicating that some somatic mutations are caused by transcription-coupled nucleotide excision repair [123]. In ASD, somatic SNVs are associated with an excess of deleterious mutations in critical exons of genes expressed during early brain development, especially those expressed in the prenatal amygdala [123]. Among the somatic SNVs, synonymous mutations tended to influence splicing, as implicated by computational prediction [12], and missense or loss-of-function mutations with allele fractions >20% were more abundant in the probands than in the siblings [11]. Notably, male siblings without ASD exhibited moderate ASD-like traits if they possessed deleterious somatic mutations with a low allele fraction [11]. Furthermore, C>T transitions were enriched in the detected somatic mutations, which Dou et al. [11] inferred were the products of 5mC deamination.

Rett syndrome, which is characterized by repetitive stereotyped movements and autistic features, is caused by mutations in *MECP2* located on the X chromosome [124]. Rett syndrome usually occurs only in females, as a lack of functional *MECP2* results in embryonic lethality. Most patients with Rett syndrome exhibit de novo germline mutations in *MECP2*, although some exhibit somatic mutations in *MECP2* in blood cells [125]. The somatic mutations should be shared between the brain and peripheral blood cells, resulting in the characteristic symptoms of Rett syndrome. Although very rare, Rett syndrome can occur in male patients, likely due to somatic mutations in *MECP2*.

## Somatic mutations in phenotypically discordant monozygotic twins

As monozygotic twins are assumed to have identical genomic information, any phenotypic discordance between two such siblings is classically attributed to environmental factors. However, monozygotic twins have different somatic mutation profiles, which may also account for phenotypic discordance. Several studies have reported monozygotic twins with a discordance of genetic diseases due to somatic chromosomal abnormalities or CNVs [126, 127]. Comprehensive analyses including WGS have identified several somatic SNVs in monozygotic twins without neuropsychiatric diseases [128, 129], although these studies did not associate somatic mutations with individual phenotypes.

Other research groups have identified somatic mutations in discordant monozygotic twins, with only the affected twin exhibiting somatic mutations in the relevant genes, in the following disorders: Darier disease (*ATP2A2*) [130], Van der Woude syndrome (*IRF6*) [131], Dravet syndrome *(SCN1A*) [132], and neurofibromatosis type I (*NF1*) [133]. One study involving exome-wide investigation detected mutations in *FBXO38*, *SMOC2*, and *TDRP* only in the affected twin from a pair of monozygotic twins discordant for gender dysphoria [134]. A previous study also reported that monozygotic twins discordant for the severity of fragile X syndrome exhibited differences in CGG triplet repeat numbers [135]. Several pairs of monozygotic twins discordant for Parkinson’s disease had discordant CNV profiles in blood cells [136]. In addition, we identified a missense somatic mutation in *ABCC9* in one twin with a delusional disorder, which was not present in the healthy cotwin [137]. A previous meta-analysis of GWAS research revealed that *ABCC9* is associated with sleep duration [138], indicating that somatic mutations of this gene may be associated with delusional disorders. Overall, studies of discordant monozygotic twins, particularly when combined with analyses of de novo and somatic mutations, will aid researchers in identifying risk genes for psychiatric disorders.

## Technical issues in psychiatric research

Several issues must be considered during analyses of somatic mutations in human brain samples: (i) the brain region, (ii) sampling method, (iii) sequencing strategy, (iv) candidate detection, and (v) validation.

### (i) Selecting the brain region for analysis

For most psychiatric disorders, the brain regions directly associated with disease etiology have yet to be identified. Therefore, there are no definitive criteria for selecting the appropriate brain regions for analysis. This is in notable contrast to the investigation of brain malformations, wherein the localization can be clearly defined. However, recent consortium-based neuroimaging studies have begun to identify the anatomical brain changes associated with psychiatric disorders at fine resolution. Large-scale MRI studies have demonstrated that significant volume reduction occurs in the hippocampus, amygdala, thalamus, and nucleus accumbens in patients with schizophrenia [139, 140]. Other studies have reported that schizophrenia is also associated with cortical thinning due to reductions in gray matter volume in regions such as the left superior temporal gyrus and left Heschl’s gyrus [141, 142]. Similarly, large-scale MRI studies have identified significant volume reduction in the hippocampus, thalamus, left pars opercularis, left fusiform gyrus, and left rostral middle frontal cortex in patients with bipolar disorder [143, 144]. While such changes are not always related to disease onset, they may be the first choice for investigating somatic mutations.

As postmortem brain tissue is not always available, peripheral tissues must be used as alternatives in many cases. Previous studies have identified causative somatic mutations in the relevant genes using peripheral tissue samples from patients with brain malformations or Rett syndrome [105, 106, 125]. Somatic mutations have also been identified in potential risk genes using blood samples from patients with ASD [11, 12, 122, 123]. The allele fraction of somatic mutations in the relevant genes ranged from 1 to 14% in the Jamuar et al. [106] study and from 1 to 43% in the Riviere et al. [105] study in peripheral tissues, indicating that these mutations likely occurred early in development and are shared between the brain and peripheral tissues. Assuming an estimated 2.8 substitution mutations per early embryonic cell per cell doubling [24], several somatic mutations occurring early in development should exist in the blood, and can be detected with high-depth sequencing. Thus, peripheral tissues may be helpful if the somatic mutations associated with the target disease occur early in development.

However, aging should be taken into account when using peripheral blood cells. Several large-scale cohort studies reported higher allele fractions and higher rates of somatic SNVs and CNVs in peripheral blood cells in older adults due to clonal expansion from hematopoiesis [145–148]. In these studies, detectable somatic mutations were in individuals aged 40 years or older but were rare in younger populations. One study involving two pairs of monozygotic twins suggested that the older pairs tended to have discordant mutations (somatic mutations), likely due to clonal expansion associated with hematopoiesis [149]. Therefore, researchers should account for clonal expansion during hematopoiesis when investigating somatic mutations in peripheral blood cells. Samples from younger individuals (under 40 years of age) are desirable to reduce the possibility of hematopoiesis-derived somatic mutations.

### (ii) Sampling method

Single-cell analysis is advantageous  for detection of somatic mutations that occur later in development. However, false positives are induced by deamination during cell lysis, amplification bias, and errors during WGA [48]. Each WGA method (MDA, MALBAC, or DOP-PCR) is associated with advantages and disadvantages [150], necessitating careful examination. Linear amplification via transposon insertion (LIANTI), a recently proposed WGA method, is suggested to reduce amplification bias [48]. False positives indicating C>T due to spontaneous C>U deamination during single-cell preparation can be reduced using uracil DNA glycosylase [48]. Somatic cell nuclear transfer (SCNT) of neuronal nuclei into enucleated oocytes has also been proposed as a method of WGA, although this method has only been applied to single neurons in mice [151]. In six mouse neurons, WGS after SCNT identified an estimated 86.2 SNVs, 22.5 insertion/deletions, 2.5 structural variants, and 1.3 mobile element insertions per neuron on an average. The authors claimed that SCNT results in fewer amplification errors than does conventional WGA, as it utilizes a natural cellular proliferation mechanism.

Analyses of bulk brain tissue are complementary to single-cell analysis, as they offer several distinct advantages; such analyses are (i) free of C>T false positives due to C>U deamination during cell lysis [48], (ii) free of PCR or WGA errors when using PCR-free library preparations, (iii) allow for approximate assessment of allele fractions, (iv) and are more applicable to clinically oriented research involving large sample sizes.

When a target population of cells has been defined, accurate methods of sorting or dissection would be useful. We sorted bulk brain tissue into neuronal and non-neuronal nuclei using the NeuN-based fluorescence-activated cell-sorting method for analysis of LINE-1 [89], and recent studies reported methods for sorting the nuclei of oligodendrocytes [152], GABAergic interneurons, and glutamatergic neurons [153] from human postmortem brains.

### (iii) Sequencing strategy

Selecting the target genomic region is among the most important issues. Although risk genes have yet to be identified for most psychiatric disorders, recent genomic studies have revealed several candidate genes as rare variants with strong effect sizes. However, if the target genes are unclear, more comprehensive methods such as WGS or WES are required. The selection of WGS, WES, or TAS depends on the research purpose as well as the target disorder. We performed sample size calculation to detect deleterious somatic mutations of early embryonic origin using WES at a depth of 300×, by assuming that deleterious somatic mutations were enriched 1.8-fold in a case group, which was a ratio similar to that of germline de novo mutations reported by Iossifov et al. [14]. This resulted in a requirement of 250 samples for each group for a statistical power of 0.8 (Supplementary Note). TAS using a molecular inversion probe [154], which has been used to detect rare germline variants in large cohorts of patients with ASD [19], would be cost effective for detecting somatic as well as germline mutations.

The choice of the sequencing machines is also important. Most current somatic mutation studies have adopted the HiSeq or MiSeq machines (Illumina, San Diego, CA, USA) [155] as massively parallel sequencers for WGS, WES, or TAS. Illumina short-read technology is cost effective, but it is difficult to analyze regions with large homologous sequences or repeat regions, including retrotransposons with this method [156]. Target enrichment sequencing methods such as RC-Seq [65, 66] and L1Hs-Seq [70] are required to analyze repeat regions. Illumina short-read sequencing is associated with other issues as well, such as index switching [157] and systematic errors [158], requiring caution before investigating somatic mutations. New sequencing technologies involving long-read sequencing such as PacBio/Sequel (Pacific Biosciences, Menlo Park, CA, USA) [159] and MinION/PromethION (Oxford Nanopore Technology, Oxford, UK) [160] would be advantageous for investigating structural variants and retrotransposons. CNV analysis can be performed using microarrays, but low-depth WGS is more sensitive for the detection of somatic CNVs [53], and long-read sequencers are advantageous for detecting somatic structural variants including CNVs.

### (iv) Candidate detection

The allele fractions of somatic mutations in neural tissue are expected to be low due to its low proliferative capacity following differentiation. This is in notable contrast to cancer, which is characterized by hyper-proliferation and is associated with somatic mutations carrying large allele fractions. Thus, a highly sensitive approach is required to detect somatic mutations in the human brain. Cibulskis et al. [161] reported the sensitivity of MuTect, a well-known somatic mutation detection tool with high sensitivity and specificity, simulating various cases of sequencing depth. For example, MuTect requires a sequence depth of 340×~ to achieve a sensitivity of 90% when detecting somatic mutations with allele fractions of 2%. MuTect [161], VarScan [162], and Strelka [163] (all three pacakages have update of version 2) are frequently used to detect somatic mutations associated with cancer by comparing target and control tissues. One comparison study reported that MuTect has the highest sensitivity and specificity among the devices tested [164].

Notably, false positives due to DNA damage, PCR errors, sequence errors, and alignment errors are included in reports of somatic mutations. False positives of G>T or C>A due to 8-oxoguanine during PCR sample preparation were abundant in human samples, especially in cancer samples [165]. Sequencing biases have also been reported using the Illumina sequencer [158]. Caution is also required during single-cell analysis due to the artificial spontaneous C>U deamination that occurs during cell lysis [48]. Huang et al. [166] proposed a somatic mutation detection pipeline that ensures careful exclusion of probable false positives, subsequently introducing a somatic mutation detection software called MosaicHunter [167], which are designed to detect somatic mutations without the use of control tissues.

### (v) Validation

The depth of sequencing is critical for reliable identification of somatic mutations. When WES data obtained from the blood samples of patients with ASD were used [11, 12, 122, 123], validation rates among the studies ranged from 50 to 90% depending on the methods and their sensitivity. These studies were not conducted at a sufficient depth to investigate somatic mutations, as the WES data were originally acquired to investigate germline mutations.

Candidates for somatic mutation must be validated using other genetic techniques such as ddPCR, pyrosequencing, or Sanger sequencing. Among these, ddPCR is advantageous in that it allows for precise calculation of the allele fraction and exhibits high-detection sensitivity (0.001%~) [168]. Pyrosequencing and Sanger sequencing with many clones can also be used to calculate the allele fraction, although the resolution and sensitivity are lower than those of ddPCR (pyrosequence, ~5%) [169]. However, all these techniques exhibit difficulty in detecting SNVs in repeat regions including retrotransposons due to difficulty in PCR. TAS at ultrahigh depth (e.g., 10,000×~) can be used as an alternative method of validation, as this method can detect somatic mutations with allele fractions of as low as 0.1%. However, there is a potential for systematic errors when using sequencing chemistry similar to that of initial sequencing. Somatic CNVs and retrotransposons in bulk tissues can be validated using qPCR, although the resolution is limited.

## Conclusion

In the present review, we discussed current genomic studies of somatic mutations in the human brain. Somatic mutations including SNVs, CNVs, and retrotransposition have been observed in the brains of humans with and without neuropsychiatric diseases. Somatic SNVs in known risk genes have been identified for several psychiatric disorders as well as brain malformations, likely contributing to disease liability. The enrichment of somatic SNVs in neuron-expressed genes indicates their relevance in neural system dysfunction. Taken together, the accumulated evidence indicates that somatic mutations may be associated with the mechanisms underlying certain psychiatric disorders, although further research is required. As few studies have demonstrated a causal relationship of somatic mutations to disease phenotypes, future studies utilizing model animals or cells are required to demonstrate the association between the development of neuropsychiatric diseases and somatic mutations.

## Electronic supplementary material

Supplementary Note
