# Supplementary material for: Somatic mutations in the human brain: implications for psychiatric research
Source: Mol Psychiatry. 2018 Aug 7;24(6):839–56. doi: 10.1038/s41380-018-0129-y (PMC6756205; doi:10.1038/s41380-018-0129-y)
Supplement: Supplementary file 1 — Supplementary Note [file 41380_2018_129_MOESM1_ESM.docx]

**Supplementary Note**

**Sample size estimation using whole exome sequencing** (**WES) in a virtual scenario**

We considered a situation with WES at a depth of 300×, for blood samples, to detect somatic mutations of early embryonic origin, assuming equally covered WES data. We considered the proportions of individuals with LGD (likely gene disrupting [e.g., nonsense, frameshift]) variants in control and case groups (LGD_ctr_proportion_ and LGD_case_proportion_, respectively) as the main parameters, and the difference in LGD_ctr_proportion_ and LGD_case_proportion_ as the effect size (Cohen's *h*). Bae et al. reported that somatic SNVs with alternative allele fractions (AAFs) of 2% or higher were shared among multiple tissues, including the brain.[^1^](#_ENREF_1) Therefore, somatic mutations of early embryonic origin with AAFs of around 2% or higher can be detected using blood samples.

The expected values of LGD variants of early embryonic origin in control and case groups are postulated as:

$$\mathrm{LGD}_{\mathrm{ctr}}=(\mathrm{SNV}_{\mathrm{somatic}}+\mathrm{INDEL}_{\mathrm{somatic}})\times Coverage\times\mathrm{LGD}_{exon\_fraction}\times Sensitivity$$

$$\mathrm{LGD}_{\mathrm{case}}=\mathrm{LGD}_{\mathrm{ctr}}\times\mathrm{Ratio}_{\mathrm{assumed}}$$

where,

LGD_ctr_: expected value of detected LGD variants in one control sample

LGD_case_: expected value of detected LGD variants in one case sample

SNV_somatic_: somatic SNVs of early-embryonic origin in whole genome, postulated as 62 (see below) × 1.3 (somatic SNV in one cell division from Bae et al[^1^](#_ENREF_1))

# AAFs of somatic variants that have occurred until the 32-cell stage is 1.56% (1/64) or higher on an average. Until the 32-cell stage, the embryo has undergone 62 cell divisions, assuming symmetrical cell divisions. Not all these somatic variants have an AAFs of 1.56% (1/64) or higher, but we assume that other somatic variants of early-embryonic origin after the 32-cell stage could compensate for the expected value.

INDEL_somatic_: somatic INDELs of early-embryonic origin in whole genome, postulated as SNV_somatic_×0.1 (similar rate to germline *de novo* variants in Besenbacher et al.[^2^](#_ENREF_2) and Iossifov et al.^3^)

Coverage: covered fraction to whole genome. 1/50 (60Mb) for exome

LGD_exon_fraction_: LGD fraction to all the *de novo* exon mutations (both SNV and INDEL), postulated as 0.1 in control (similar rate to germline exon variants in Iossifov et al.[^3^](#_ENREF_3))

Sensitivity: Sensitivity of sequencing and informatics to all the variants of early-embryonic origin, postulated as 0.85 from MuTect simulation data[^4^](#_ENREF_4)

Ratio_assumed_: Cases-to-control ratio of LGD variant occurrence in target genomic regions (all exons): We assume three parameters, 1.4 (lower assumption) , 1.8 (similar ratio to germline de novo mutations in Iossifov et al.[^3^](#_ENREF_3)) and 2.2 (higher assumption)

Assuming that the occurrence of LGD variants follows Poisson distribution with the average rate of LGD_ctr_ (0.15, calculated as above) or LGD_case_, the proportions of individuals having one or more LGD variants in control and case groups (LGD_ctr_proportion_ and LGD_case_proportion_, respectively) are:

$$\mathrm{LGD}_{{ctr\_proportion}}=P\left( X\geq1 \right){=\sum_{k=1}^{\infty} e^{-\mathrm{LGD}_{\mathrm{ctr}}}\frac{{\mathrm{LGD}_{\mathrm{ctr}}}^{k}}{k!}}$$

$$=1-P(X=0){=1-e^{-\mathrm{LGD}_{\mathrm{ctr}}}\frac{{\mathrm{LGD}_{\mathrm{ctr}}}^{0}}{0!}}$$

$$\mathrm{LGD}_{{case\_proportion}}=P\left( X\geq1 \right){=\sum_{k=1}^{\infty} e^{-\mathrm{LGD}_{\mathrm{case}}}\frac{{\mathrm{LGD}_{\mathrm{case}}}^{k}}{k!}}$$

$$=1-P(X=0){=1-e^{-\mathrm{LGD}_{\mathrm{case}}}\frac{{\mathrm{LGD}_{\mathrm{case}}}^{0}}{0!}}$$

where,

k: number of LGD variant in one individual

P(X=k): one individual’s probability of having k LGD variant calculated by Poisson’s distribution

Cohen’s *h*, required sample size (*N*) and odds ratio (OR) are calculated with the functions in "pwr" R package (https://cran.r-project.org/web/packages/pwr/index.html) as:

$$Cohen’s h=ES.h\left( \mathrm{LGD}_{case\_proportion}, \mathrm{LGD}_{ctr\_proportion} \right)$$

$$N=pwr.2p.test(h=h,sig.level=0.05, power=0.8, alternative="greater")$$

$$OR=\frac{\mathrm{LGD}_{case\_proportion}\times(1-\mathrm{LGD}_{ctr\_proportion})}{\mathrm{LGD}_{ctr\_proportion}\times(1-\mathrm{LGD}_{case\_proportion})}$$

*N*: required number of observations in each group for statistical power of 0.8, setting type I error probability as 0.05

The results of the calculations are as follows:

| Ratio_assumed_  (LGD_case_/LGD_ctr_) | Cohen's *h* | Required observations (*N* for each group) | OR |
| --- | --- | --- | --- |
| 1.4 | 0.104 | 1140 | 1.32 |
| 1.8 | 0.222 | 250 | 1.76 |
| 2.2 | 0.322 | 120 | 2.21* |

*similar to average OR of de novo LGD variants in WES for ASD[^5^](#_ENREF_5)

The relationship between Cohen’s *h* and required sample size (*N*) for each statistical power (0.4 to 0.9) is plotted in Figure S1.

Figure S1. Sample size estimation for different statistical power


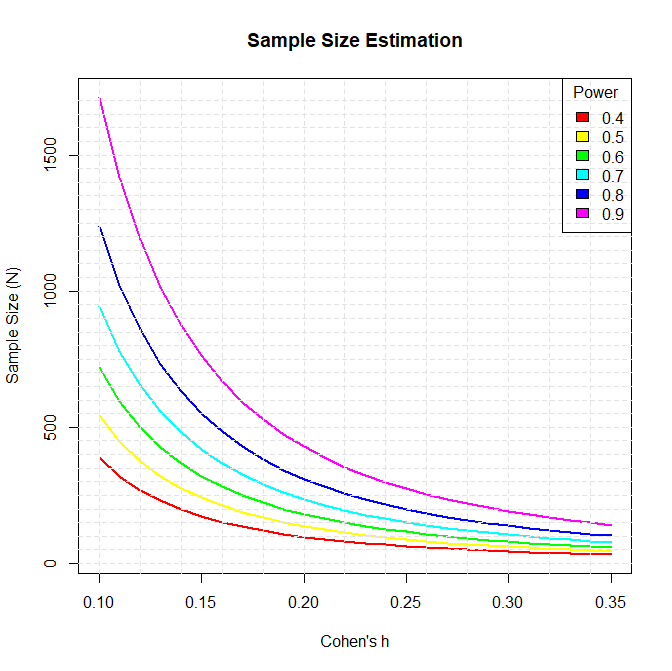


Supplementary Reference

1. Bae T, Tomasini L, Mariani J, Zhou B, Roychowdhury T, Franjic D *et al.* Different mutational rates and mechanisms in human cells at pregastrulation and neurogenesis. *Science* 2017.

2. Besenbacher S, Liu S, Izarzugaza JM, Grove J, Belling K, Bork-Jensen J *et al.* Novel variation and de novo mutation rates in population-wide de novo assembled Danish trios. *Nat Commun* 2015; **6:** 5969.

3. Iossifov I, O'Roak BJ, Sanders SJ, Ronemus M, Krumm N, Levy D *et al.* The contribution of de novo coding mutations to autism spectrum disorder. *Nature* 2014; **515**(7526)**:** 216-221.

4. Cibulskis K, Lawrence MS, Carter SL, Sivachenko A, Jaffe D, Sougnez C *et al.* Sensitive detection of somatic point mutations in impure and heterogeneous cancer samples. *Nat Biotechnol* 2013; **31**(3)**:** 213-219.

5. Robinson EB, Neale BM, Hyman SE. Genetic research in autism spectrum disorders. *Curr Opin Pediatr* 2015; **27**(6)**:** 685-691.
